# Charge Storage and Reliability Characteristics of Nonvolatile Memory Capacitors with HfO_2_/Al_2_O_3_-Based Charge Trapping Layers

**DOI:** 10.3390/ma15186285

**Published:** 2022-09-09

**Authors:** Dencho Spassov, Albena Paskaleva, Elżbieta Guziewicz, Wojciech Wozniak, Todor Stanchev, Tsvetan Ivanov, Joanna Wojewoda-Budka, Marta Janusz-Skuza

**Affiliations:** 1 Institute of Solid State Physics, Bulgarian Academy of Sciences, Tzarigradsko Chaussee 72, 1784 Sofia, Bulgaria; 2 Institute of Physics, Polish Academy of Sciences, Al. Lotników 32/46, 02-668 Warsaw, Poland; 3 Institute of Metallurgy and Materials Science, Polish Academy of Sciences, ul. Reymonta 25, 30-059 Cracov, Poland

**Keywords:** nonvolatile memory, charge trapping, atomic layer deposition (ALD), HfO_2_/Al_2_O_3_ nanolaminates, Al-doped HfO_2_, TEM characterization

## Abstract

Flash memories are the preferred choice for data storage in portable gadgets. The charge trapping nonvolatile flash memories are the main contender to replace standard floating gate technology. In this work, we investigate metal/blocking oxide/high-k charge trapping layer/tunnel oxide/Si (MOHOS) structures from the viewpoint of their application as memory cells in charge trapping flash memories. Two different stacks, HfO_2_/Al_2_O_3_ nanolaminates and Al-doped HfO_2_, are used as the charge trapping layer, and SiO_2_ (of different thickness) or Al_2_O_3_ is used as the tunneling oxide. The charge trapping and memory windows, and retention and endurance characteristics are studied to assess the charge storage ability of memory cells. The influence of post-deposition oxygen annealing on the memory characteristics is also studied. The results reveal that these characteristics are most strongly affected by post-deposition oxygen annealing and the type and thickness of tunneling oxide. The stacks before annealing and the 3.5 nm SiO_2_ tunneling oxide have favorable charge trapping and retention properties, but their endurance is compromised because of the high electric field vulnerability. Rapid thermal annealing (RTA) in O_2_ significantly increases the electron trapping (hence, the memory window) in the stacks; however, it deteriorates their retention properties, most likely due to the interfacial reaction between the tunneling oxide and the charge trapping layer. The O_2_ annealing also enhances the high electric field susceptibility of the stacks, which results in better endurance. The results strongly imply that the origin of electron and hole traps is different—the hole traps are most likely related to HfO_2_, while electron traps are related to Al_2_O_3_. These findings could serve as a useful guide for further optimization of MOHOS structures as memory cells in NVM.

## 1. Introduction

Nonvolatile memories (NVMs) are an integral and very important part of advanced electronic systems such as smartphones and handheld devices because they offer a small, low-power-consuming, and reliable alternative to disk storage. The rapid increase in memory density along with the cost reduction have resulted in an ever-growing segment of the NVM memories market. Up until now, the dominant NAND flash NVM technology was the floating gate memory cell in which the charge is stored in an electrically isolated poly-Si gate [1,2]. However, the increasing demands for larger volumes of stored data have caused an aggressive down-scaling of cell sizes and, consequently, the intrinsic limitations of floating gate technology have become insurmountable. Charge trapping (CT) NVMs are considered a promising alternative to the conventional floating gate technology as they offer better operation characteristics, e.g., improved retention and endurance, lower power consumption, and higher program/erase (P/E) speed [3,4,5]. Moreover, the usage of CT-NVM seems unavoidable in Vertical-NAND flash memory technology [6]. The conventional charge trapping memory cell consists of a charge trapping layer (CTL) sandwiched between two oxide layers with larger bandgaps to prevent the leakage of trapped charge, i.e., metal electrode—blocking oxide (BO)—CTL—tunneling oxide (TO)—Si structures. The charge storage in discrete, spatially isolated traps in CTLs is a significant advantage as it prevents the leakage of the stored charge through a defect path in the tunnel oxide (TO). In addition, the CT-NVM technology is fully compatible with the floating gate and CMOS technology. The first CT-NVMs were realized by using Si_3_N_4_ as charge storage media and SiO_2_ as blocking and tunneling oxides [7,8]. The use of dielectrics with a higher dielectric constant (high-k dielectrics) as an alternative to Si_3_N_4_ attracts a lot of attention because they also provide higher trap densities and larger conduction band offsets with respect to TO, which may result in improved P/E efficiencies and vertical scaling as well as larger memory windows [9,10]. HfO_2_ as the most studied high-k dielectric has been considered also for application in CT-NVMs because it is a trap-rich material, and the study of You et al. [11] revealed that the 2 nm HfO_2_ layer has a better charge trapping efficiency than 7 nm Si_3_N_4_. Charge trapping properties of HfO_2_ could be substantially enhanced by doping with Al atoms or stacking with Al_2_O_3_ [12,13,14,15,16]. The storage characteristics could be further boosted by proper treatments, e.g., annealing steps and UV irradiation [14,17,18]. The main purpose of the doping/treatment is to modify the density as well as spatial and energy location of electrically active traps in such a way that more efficient and stable charge storage is obtained. In this context, it is very important that the trapping sites in CT-NVMs are deep enough. In our previous work [14], we have shown that doping of HfO_2_ with the proper amount of Al introduces deep Al_2_O_3_-related traps, which effectively increase the trapping ability of the stacks. Therefore, the substantial knowledge on the HfO_2_ properties and the possibility to control to some extent the density and energy position of traps, the maturity of HfO_2_ deposition (in particular, atomic layer deposition), and their full compatibility with the CMOS technology are significant advantages over the other charge-trapping alternatives, e.g., nanoparticle CT layers, which may require materials and processing (including high-thermal-budget processes) that are not CMOS-compatible. In a number of works [19,20,21], we have studied the dielectric and electrical properties of Al_2_O_3_/HfO_2_ multilayer stacks deposited by atomic layer deposition (ALD). Samples of various compositions (different thickness of Al_2_O_3_ and HfO_2_ layers and the number of Al_2_O_3_/HfO_2_ bi-layer repetitions) subjected to a post-deposition annealing (PDA) in different ambients have been investigated and assessed from the viewpoint of their potential implementation as CT layers in NVMs. It has been established that charge trapping properties could be tailored by optimization of the stack parameters as well as annealing steps. Annealing in an oxygen ambient most strongly affects the charge storage ability of the stacks by increasing the number of trapped electrons and improving the stability of the stacks to a high electric field. Moreover, we have shown that Al_2_O_3_/HfO_2_ stacks after RTA in O_2_ are radiation-tolerant and their charge storage characteristics are not deteriorated by γ-irradiation [22].

Next to CTL, it is also very important to optimize blocking and tunneling oxides and high-k dielectrics have also been considered for this purpose. The BO layer should form a potential barrier of sufficient height and thickness between the CTL and the gate electrode in order to reduce undesirable movement of electrical charges (holes/electrons) toward the gate electrode. A thin tunnel oxide is inserted between the charge-storing dielectric and the Si substrate to better control the injection process of the carriers, as well as to improve the retention characteristics. Hence, both the blocking and tunnel oxide should have a wide bandgap E_g_, so SiO_2_ with its E_g_ of about 9.1 eV is the most widely used. However, direct tunneling current through the thin tunnel SiO_2_ layer could compromise the retention characteristics. Al_2_O_3_ is the natural choice to replace SiO_2_ both as BO and TO because it has the largest bandgap (more than 8 eV) among the high-k dielectrics [23]. In addition, it has good chemical as well as thermal stability and it is CMOS-compatible. Several studies have revealed that using Al_2_O_3_ as BO could result in an improvement of memory window, retention parameters, and P/E efficiency and can mitigate a specific problem of erase saturation [24,25,26]. Agrawal et al. [27] demonstrated an all-AlO_x_ CT-NVM stack with good retention properties, where BO, TO, and CTL are AlO_x_ layers with different thicknesses and oxygen contents in the film, which was engineered by different gas ratios and pulse times of the ALD process. The use of Al_2_O_3_ as a tunnel oxide enables the entire dielectric structure of a memory cell to be obtained in a single ALD deposition process.

In this work, we study the charge trapping, retention, and endurance characteristics in metal/blocking oxide/high-k charge trapping layer/tunnel oxide/Si (MOHOS) structures. Two different HfO_2_/Al_2_O_3_-based CTL and SiO_2_ or Al_2_O_3_ tunneling oxides are used in MOHOS stacks. The effect of rapid thermal annealing in O_2_ on the operation of MOHOS stacks is also studied.

## 2. Materials and Methods

**Figure 1:**
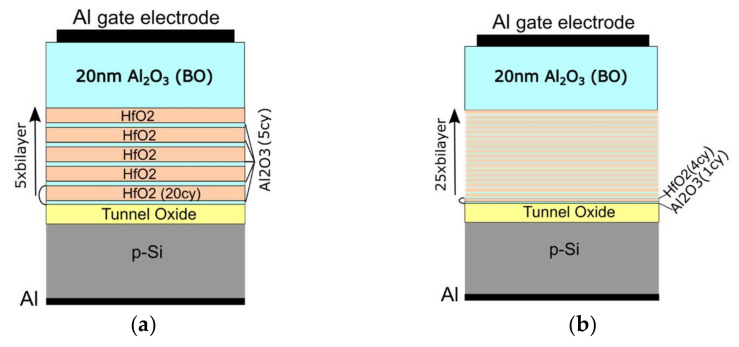
Schematic presentation of the memory capacitors with: (**a**) nanolaminated and (**b**) doped charge trapping layer.

Metal electrode/blocking oxide (BO)/high-k dielectric/tunnel oxide (TO)/semiconductor (p-Si) (MOHOS) structures were prepared for implementation as a memory cell in charge trapping memory (CTM). The high-k dielectric charge trapping layers were prepared by the thermal atomic layer deposition (ALD) method. Two types of dielectric stacks consisting of HfO_2_ and Al_2_O_3_ were made. The first type of HfO_2_/Al_2_O_3_ stack was constructed by a 5-fold repeating of a HfO_2_/Al_2_O_3_ block composed of a 20 cycles-thick HfO_2_ sublayer and 5 cycles-thick Al_2_O_3_ sublayer (Figure 1a). (The thicknesses of the building sublayers are given by the number of ALD cycles used for deposition, which is a standard practice for this technology.) These structures were briefly assigned as 5 × (20:5). In the second type of HfO_2_/Al_2_O_3_ stack, in order to achieve a doping effect, the structure of the HfO_2_/Al_2_O_3_ block was changed by reducing the thickness of the sublayers—the Al_2_O_3_ sublayer was reduced only to 1 cycle, and the HfO_2_ sublayer was 4 cycles. The number of HfO_2_/Al_2_O_3_ blocks was increased to 25 to ensure equal thickness for both types of HfO_2_/Al_2_O_3_ stacks (structures were briefly assigned as 25 × (4:1)) (Figure 1b). Depositions of HfO_2_ and Al_2_O_3_ were performed at 135 °C by using a tetrakis (dimethylamido) hafnium (TDMA) precursor and trimethylaluminum precursor (TMA), respectively. In both processes, H_2_O was used as an oxidant and nitrogen was used as a carrier and purging gas between cycles. The deposition of the HfO_2_/Al_2_O_3_ stack was followed by the deposition of a blocking Al_2_O_3_ oxide with a thickness of 200 cycles (about 20 nm).

Two types of tunnel oxide were used: (i) SiO_2_ (with two thicknesses 2.4 or 3.5 nm) grown by a standard thermal oxidation of Si; (ii) Al_2_O_3_ layer (about 3 nm thick) deposited under the same ALD conditions as mentioned above. After the deposition of dielectric layers, a part of the structures was subjected to RTA at 800 °C, for 1 min in O_2_. 

The formation of a memory cell in the form of a MIS capacitor was realized by depositing the top and bottom Al electrodes by thermal evaporation. Square top contacts were defined by photolithography.

Nanolaminated Al_2_O_3_/HfO_2_ stacks before and after annealing were subjected to detailed microstructural observations using a TECNAI G2 SuperTWIN FEG (200 kV; Thermo Fisher Scientific Inc., Waltham, MA, USA) transmission electron microscope (TEM). Characterization was carried out in TEM bright-field (TEM BF), high-resolution (HRTEM), as well as scanning-transmission TEM (STEM) modes. Chemical analyses were performed by X-ray energy-dispersive spectroscopy (EDS). Thin foils for TEM analysis were prepared by the focused ion beam (FIB) technique using a QUANTA 200 3D DualBeam microscope (Thermo Fisher Scientific Inc., Waltham, MA, USA). The charge-trapping in the stacks was evaluated by applying square negative and positive voltage pulses of different amplitudes *V*_p_ with a duration of 1 s to the top metal electrode (back Al electrode is grounded). Each pulse was followed by a measurement of the C-V curve, and its flat-band voltage, *V_fb_*, shift with respect to the initial C-V curve was determined. The memory windows were defined as the difference between the voltage shifts corresponding to the negative and positive pulses (Supplementary Figure S1). The end values of *V*_p_ were defined by the electrical breakdown of capacitors. Retention characteristics were obtained by monitoring the charge loss over time through control C-V measurements after initial charging of the capacitors with a negative or positive charge by applying a voltage pulse *V*_p_ = ±27 V, i.e., setting the capacitors to the Program/Erase state. The endurance characteristics were acquired at *V*_p_ = ±25 V, and a control C-V curve was recorded after each pulse in order to find the flat-band voltage shift.

## 3. Results and Discussion

### 3.1. Transmission Electron Microscopy (TEM)

**Figure 2:**
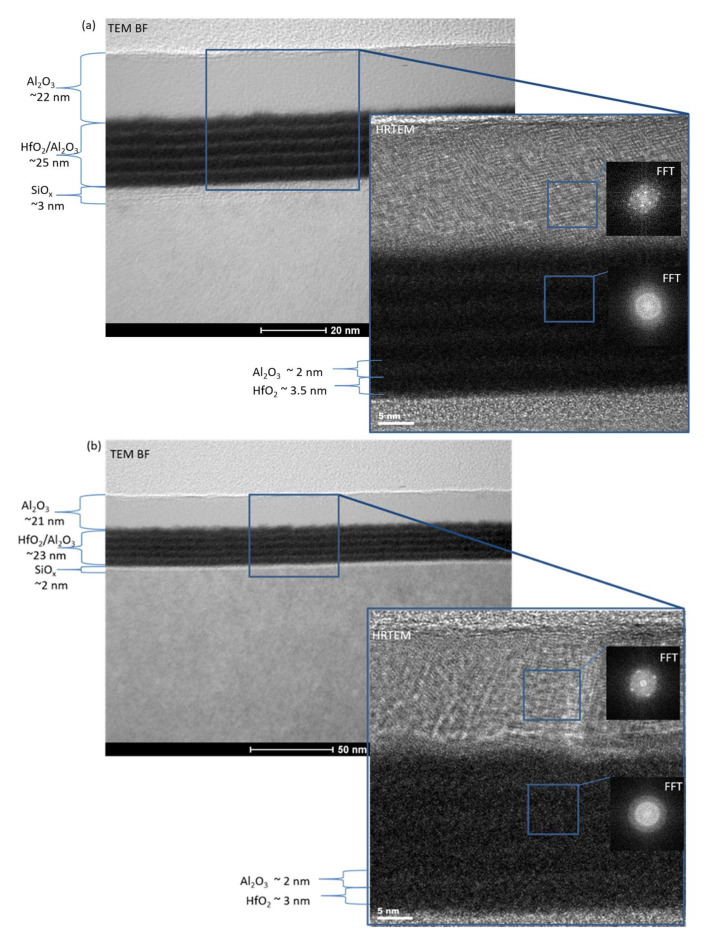
TEM characterization of nanolaminate 5 × (20:5) HfO_2_/Al_2_O_3_ high-k dielectric stack with 2.4 nm SiO_2_ and *BO* (Al_2_O_3_) before (**a**) and after O_2_ annealing (**b**).

The TEM observations (Figure 2) show a nanolaminated Al_2_O_3_-HfO_2_ 5 × (20:5) stack with SiO_2_ TO and Al_2_O_3_ BO before (Figure 2a) and after annealing (Figure 2b). The analyses confirm the multilayered structure of the Al_2_O_3_-HfO_2_ 5 × (20:5) stack. Before annealing, the thicknesses of the individual parts are estimated to be about: 2 nm—SiO_2_ TO, 25 nm—the HfO_2_/Al_2_O_3_ stack, and 22 nm—the Al_2_O_3_ BO (Figure 2a). After RTA, both thicknesses decrease to 23 nm for the HfO_2_/Al_2_O_3_ stack and to 21 nm for Al_2_O_3_ BO (Figure 2b), i.e., O_2_ annealing results in densification of the layers. As is seen, the multilayer structure is maintained after the annealing process, but the HfO_2_/Al_2_O_3_ interfaces are not so sharp as before. Obviously, some intermixing between HfO_2_ and Al_2_O_3_ layers occurs at the interface. This intermixing is very pronounced at the interface with the Al_2_O_3_ blocking layer, the roughness of which also increases after RTA. It should be mentioned that HRTEM observations (Figure 2a,b) reveal that crystallization processes of the Al_2_O_3_-HfO_2_ layers do not occur and these layers remain amorphous both before as well as after the high-temperature annealing. The analysis also shows that the Al_2_O_3_ BO part both before and after RTA has a crystal structure.

**Figure 3:**
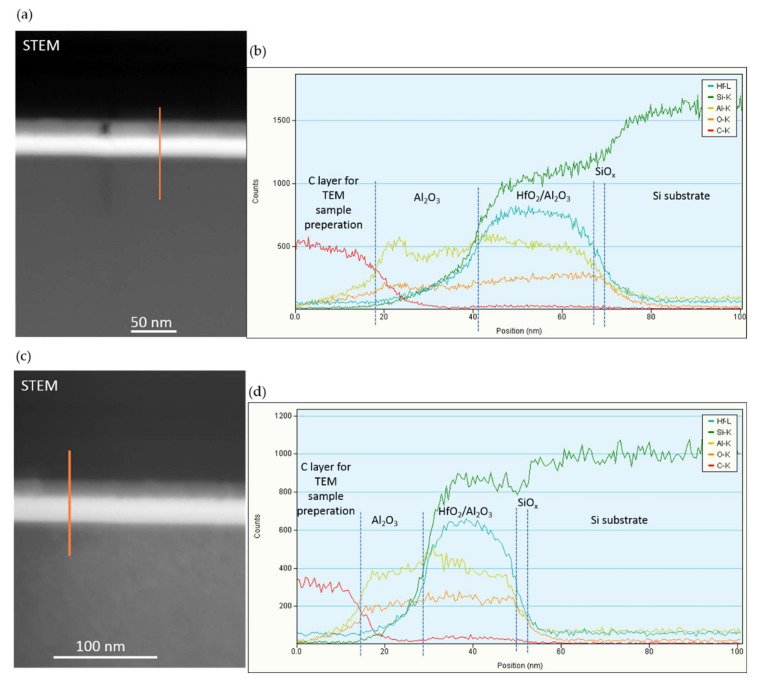
Qualitative chemical analysis (EDS) of nanolaminate 5 × (20:5) HfO_2_—Al_2_O_3_ layers with 2.4 nm SiO_2_ and *BO* (Al_2_O_3_); STEM images after (**a**) and before annealing (**c**) with marked line-scan; diagrams of the elements distributions (**b**–**d**) recorded along marked line-scan.

The EDS measurements performed along the line-scan confirm the chemical composition of the HfO_2_/Al_2_O_3_ multilayer structure (Figure 3). The results also show a reduction in the thickness of the Al_2_O_3_ BO layer and the HfO_2_/Al_2_O_3_ stack after annealing and the blurring of the HfO_2_/Al_2_O_3_ interfaces, which is evidently visible in the Hf-L distribution diagram. RTA in O_2_ does not lead to a significant increase in the concentration of oxygen in the structures but improves the homogeneity of its distribution. Another interesting feature is the change in the Si-K profile after RTA associated with the possible diffusion of Si into the HfO_2_/Al_2_O_3_ stack.

### 3.2. Charge Trapping Characteristics

**Figure 4:**
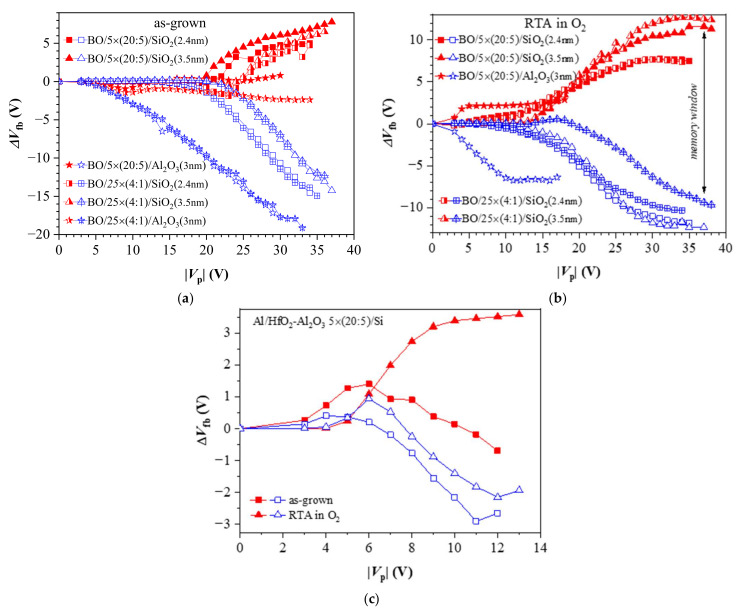
The flat band voltage shifts as a function of voltage pulse amplitude: (**a**) as-grown stacks; (**b**) after O_2_ annealing; (**c**) 5 × (20:5) HfO_2_/Al_2_O_3_ stack without any TO and *BO*. Red closed symbols correspond to +*V*_p_ and the blue open symbols to –*V*_p_.

Figure 4 compares the charge capture in nanolaminate (5 × (20:5)) and doped (25 × (4:1)) dielectric structures before and after O_2_ annealing. Before annealing (Figure 4a), the capture of positive charge in samples with Al_2_O_3_ TO starts at low *V*_p_~5 V, while for samples with SiO_2_ TO, this process occurs at substantially larger *V*_p_~16 V for 2.4 nm SiO_2_ and ~22 V for the 3.5 nm one. The flat-band voltage shift Δ*V_fb_* due to positive charge trapping increases progressively (almost linearly) with *V*_p_ and it reaches very large values of about 15–20 V with no tendency for saturation. Such a behavior has also been observed for as-deposited stacks without any TO and BO [19] and has been explained with the generation of stress-induced positively charged defects (which is an irreversible process), which adds to the hole trapping (which is a reversible process). The results also reveal that the positive charge trapping is almost the same in samples with the same TO irrespective of the dielectric stack. In other words, it depends on the tunnel oxide (and its thickness) and is weakly affected by the dielectric stack. The last result suggests that the hole capture takes place in traps associated with HfO_2_. Structures with the thinner SiO_2_ demonstrate larger hole trapping, which is explained with the larger number of injected charges due to the higher electric field in the thinner layers (at the same applied external voltage) and also to the operating charge transport mechanisms in TO (direct tunneling in the case of 2.4 nm SiO_2_ and Fowler–Nordheim tunneling for thicker SiO_2_). However, the capture of electrons depends on the dielectric, as the nanolaminate structures show a stronger electron trapping (respectively, the memory window is larger), which is evidence that this trapping occurs in Al_2_O_3_-associated traps. In addition, the electron trapping in nanolaminated stacks starts at lower charging voltages *V*_p_, which allows operation at lower electric fields. It should be noticed that Δ*V_fb_* due to negative charge trapping is significantly smaller than that due to positive charge trapping. This could be again explained by the occurrence of two competing process, which give rise to Δ*V_fb_*—trapping of electrons in existing traps and the generation of positive charge by high-field electric stress. For structures with Al_2_O_3_ TO, regardless of the dielectric layer, the negative charge trapping is very weak, which makes these structures unsuitable as a memory cell in CTM. For this reason, subsequent research is focused on structures with SiO_2_ as a tunnel oxide.

After O_2_ annealing, the trapping characteristics change notably (Figure 4b). The capture of electrons in all studied structures increases significantly compared to the negative charge trapping before RTA. This capture is similar for the stacks of the same SiO_2_ thickness and is only slightly affected by the type of dielectric stack. Compared to the stacks before annealing, the positive charge trapping decreases and exhibits a saturation at a higher *V*_p_. This behavior implies that, after RTA in O_2_, the stacks are more resistant to high-electric-field degradation and no positive charge is generated. As a result, the net positive charge trapping decreases and the net negative charge trapping increases. A similar effect is also observed in the nanolaminated stack without any TO and BO (Figure 4c), as well as in stacks with various HfO_2_/Al_2_O_3_ ratios without BO and TO [19]. This is most likely the reason why the two branches of the trapping characteristics become more symmetrical. In contrast to pre-annealing structures where hole trapping does not depend on CTL (nanolaminate or doped stack) (Figure 4a), after RTA, the hole trapping is stronger in nanolaminate structures (Figure 4b). The electron and hole trappings start at lower *V*_p_ compared to as-deposited stacks, which means lower operation voltages. It should be noted that the nanolaminated 5 × (20:5) stack with Al_2_O_3_ TO exhibits an increase in negative charge trapping and a significant memory window (about 9 V at *V*_p_ > 10 V) forms. However, the positive and negative charge trappings are strongly asymmetric, and a major part of the memory window (about 7 V) is due to positive charge trapping. The doped 25 × (5:1) stack with Al_2_O_3_ TO does not show any charge trapping (neither positive nor negative) after RTA and thus is not shown in Figure 4b.

The density of trapped electrons and holes was estimated using its relation to Δ*V_fb_* [28]:

(1) $$\Delta V_{fb}=\frac{q\rho X_{T}}{\varepsilon_{BO}}\left(d_{BO}+\frac{\varepsilon_{BO}X_{T}}{2\varepsilon_{T}}\right)$$

where *ρ* is the spatial density of the trapped charge, *q* is the charge of the electron, *ε_BO_* is the dielectric constant of *BO*, *d_BO_* is the thickness of *BO*, and *X_T_* and *ε_T_* are the thickness and dielectric constant of the charge trapping layer, respectively. *ρ* for electrons and holes is evaluated only for the annealed samples, as the observed saturation of Δ*V_fb_* at high *V*_p_ suggests that all available traps are filled, so *ρ* represents the density of the traps. The obtained values are presented in Table 1.

### 3.3. Retention Characteristics

#### 3.3.1. Structures before O_2_ Annealing

**Figure 5:**
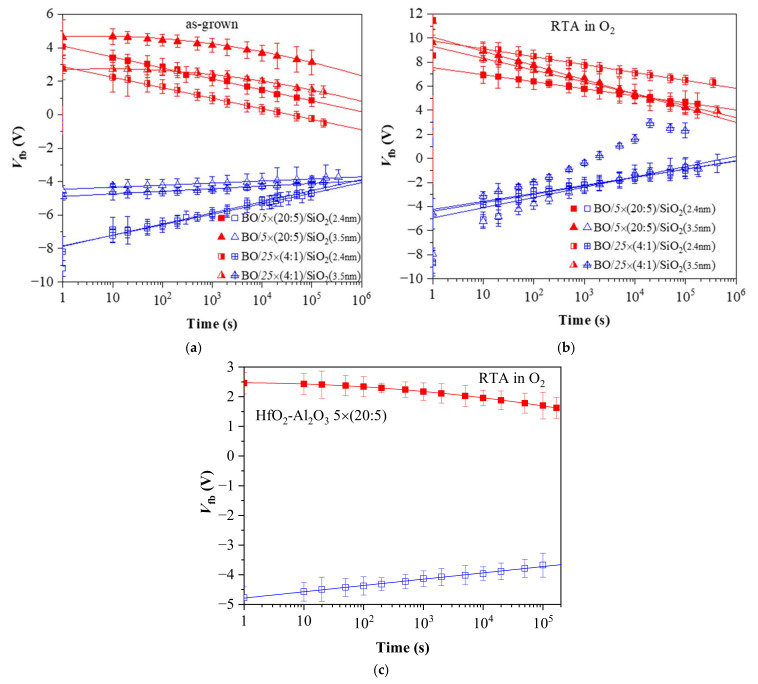
Charge retention characteristics in capacitors with various HfO_2_-Al_2_O_3_ stacks: (**a**) before and (**b**) after RTA in O_2_; (**c**) HfO_2_-Al_2_O_3_ 5 × (20:5) stack without *BO* and TO after RTA. The filled red symbols correspond to a negative charge (respectively, positive values of *V_fb_*) and the open blue ones correspond to a positive charge (negative values of *V_fb_*).

The retention characteristics measured after a pulse voltage of *V*_p_ = 27 V for structures before and after O_2_ annealing are presented in Figure 5. The results show that the retention characteristics of holes for the 5 × (20:5) and 25 × (4:1) structures before RTA almost coincide for the same thickness of the tunnel SiO_2_ (Figure 5a), i.e., the retention of holes in the structures depends on the TO thickness and is almost independent of the dielectric stack (nanolaminate structure or doped oxide). The retention characteristics also confirm the stronger positive charge trapping in structures with a thinner 2.4 nm SiO_2_ irrespective of the dielectric layer. The discharge of trapped holes follows a linear law in the log(*t*) scale, which is consistent with the trap-to-band tunneling mechanism (from the trap level in high-k stack through the SiO_2_ layer to the Si valence band). For this detrapping process, the time constant of the hole is given as τ_0_∙exp(α_TO_d_TO_)exp(α_high-k_X), where τ_0_ is a constant for the traps; *d*_TO_ is the thickness of TO; *X* is the trap distance from TO (tunneling distance); α_TO_ and α_high-k_ are coefficients dependent on trap energy and band offsets [29]. As can be seen, the observed discharge rate is higher for stacks with thinner SiO_2_ that correlates with the higher probability of back-tunneling of charge carriers in this case. 

The discharge rate of holes through 3.5 nm SiO_2_ is very low, i.e., SiO_2_ of such a thickness provides a good barrier to back-tunneling of holes. Neither charge trapping nor the discharge mechanism and rate depend on the dielectric stack—nanolaminated HfO_2_/Al_2_O_3_ or Al-doped HfO_2_. These results imply that the hole trapping in the two kinds of stacks occurs in the same type of traps, i.e., these traps most likely are related to HfO_2_. The tunneling SiO_2_ layer dominates the retention characteristics of holes (discharge rate) by spatially shifting the traps position further from Si and forming an additional potential barrier.

Figure 5a also confirms the stronger electron trapping in the HfO_2_/Al_2_O_3_ multilayer structures compared to Al-doped HfO_2_ ones. In the case of electrons as well as holes, the discharge rate is higher for structures with a thinner tunnel SiO_2_ and it is evident that electron detrapping follows different discharge laws for the samples with 2.4 and 3.5 nm SiO_2_. For capacitors with a 2.4 nm tunnel SiO_2_, the *V_fb_* dependence is linear in the log(*t*) scale, i.e., the electron discharge performs through the trap-to-band tunneling mechanism. It should be noted that the straight lines for the 5 × (20:5) and 25 × (4:1) stacks are parallel, which implies the same discharge mechanism for both stacks. For structures with a 3.5 nm tunnel SiO_2_, the *V_fb_* dependence on log(*t*) is more complicated in the case of electron discharge, which suggests that either the mechanism of electron loss is different or several mechanisms operate in parallel. More specifically, the voltage decay is well fitted by the ln^2^(*t*) dependence, indicating that the electron detrapping most likely occurs via the Poole–Frenkel mechanism [19,30]. Therefore, for a stored negative charge, field-assisted thermal excitation of the trapped electrons takes place and appends to the charge loss determined by tunneling. As the contribution of PF emission is not clearly observed for the trapped holes, we could suggest that hole traps are much deeper than the electron ones. This could be qualitatively inferred from the fact that for a given electric field distribution in the stack (i.e., distribution of the trapped charge), the electric-field-induced barrier lowering is the same for all kind of traps. However, in case of deep traps, this lowering is not enough for sizable thermal excitation of the trapped carriers at room temperature, i.e., for PF emission. For the shallow enough traps, on the other hand, the electric field provides enough barrier reduction so that carriers could be readily emitted to the corresponding band at room temperature; hence, the PF mechanism is observed. It should also be noted that the shape of the *V_fb_* vs. log(*t*) dependence could also reflect the spatial and energy distribution of the traps. In any case, however, different *V_fb_* vs. log(*t*) dependencies for trapped electrons and holes support the assumption that the origin of these centers is different. The electron discharge in stacks with 3.5 nm SiO_2_ does not depend on the type of dielectric stack as well, as the curves for the two dielectrics are parallel to each other. These results allow the conclusion that electron traps in the two kinds of stacks have the same origin, but their density is higher in multilayered 5 × (20:5) stacks.

#### 3.3.2. Structures after O_2_ Annealing

After O_2_ annealing, significant changes occur not only in the number of trapped charges (electrons or holes), but also in the discharge characteristics and their dependence on the parameters of the structure (thickness of TO and the type of dielectric stack) (Figure 5b). For samples with a thinner 2.4 nm SiO_2_ TO, despite the stronger electron trapping and weaker hole trapping after O_2_ annealing, the discharge rates of both types of charges are similar to those in the structures before O_2_ annealing. In other words, oxygen annealing changes the density of the trapped charge, but does not alter their discharge mechanism in stacks with 2.4 nm SiO_2_.

Contrary to expectations, the electron discharge rate is higher in structures with a thicker 3.5 nm SiO_2_ compared to stacks with a thinner 2.4 nm SiO_2_ and slightly depends on the dielectric stack. This result is unexpected and shows that the annealing most likely results in a reaction between SiO_2_ and the dielectric, which leads to the formation of traps in the TO, through which the stored charge is discharged. The discharge rate of the trapped holes is also higher than that in the structures before O_2_ annealing, with the highest being for doped layers (25 × (5:1)) on thicker SiO_2_. These results show that although O_2_ annealing leads to strong electron trapping in the dielectric layer, it is not suitable for structures with TO and *BO*, as it generates defects (most likely due to the interaction between the HfO_2_/Al_2_O_3_ charge trapping layer and TO), which cause a faster discharge of the charges stored in the structures. This finding is in line with reports of other authors [31] that high-temperature annealing deteriorates the retention properties of tunneling oxide. Further, as it might be inferred from Figure 5b, the discharge rate within first 10 s of the annealed stacks is noticeably higher than that of the corresponding as-grown structures, which also agrees well with the suggested defect generation in TO and the high-*k*/TO interface during the annealing. This conclusion is additionally supported by the retention characteristics of the O_2_-annealed multilayered 5 × (20:5) stacks without any intentionally grown TO and *BO* (Figure 5c). As is seen, the hole discharge follows a linear law, while electron discharge is well fitted by ln^2^(*t*), i.e., the retention in these stacks is very similar to the retention in stacks with a thicker SiO_2_ TO and *BO* before annealing (Figure 5a).

### 3.4. Endurance Characteristics

**Figure 6:**
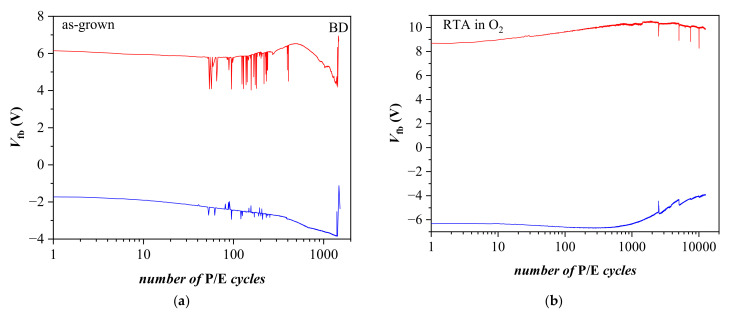
Endurance of *BO*/5 × (20:5)/TO (3.5 nm SiO_2_) capacitors before (**a**) and after O_2_ annealing (**b**) measured under voltage pulses +/−25 V. Red lines correspond to *V*_p_ = 25 V, blue ones to *V*_p_ = −25 V.

**Figure 7:**
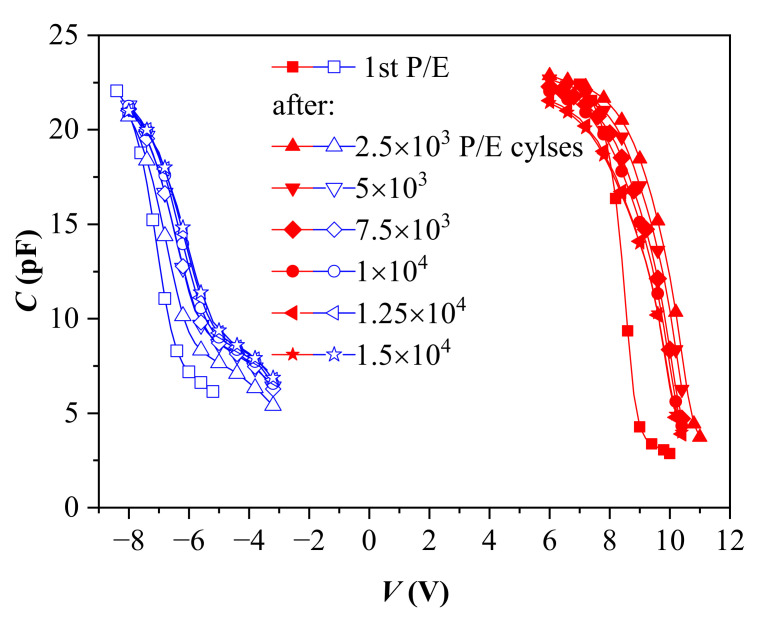
C-V curves during the endurance measurements with ±25 V voltage pulses.

The endurance characteristics measured at pulse voltage *V*_p_ = ±25 V of as-deposited and annealed nanolaminated HfO_2_/Al_2_O_3_ stacks with 3.5 nm SiO_2_ TO are shown in Figure 6. During the first several hundred program/erase (P/E) cycles, the as-deposited structure (Figure 6a) reveals some instabilities, especially in electron trapping, which tend to recover. However, after about 600 P/E cycles, substantial degradation in both positive and negative charge trapping is observed, with a stable tendency to increase positive charge trapping and decrease negative charge trapping. Such a behavior could be explained by the progressive accumulation of positive charge generated by the high electric field, i.e., it is fully consistent with the trapping characteristics in Figure 4a. It should be mentioned that the annealed samples reveal better endurance than as-deposited ones. As is seen in Figure 6, the former structures can withstand more than 10^4^ P/E cycles without coming to breakdown, while the as-deposited structures are broken-down shortly after 1000 P/E cycles. In addition, the endurance characteristics of the annealed sample are qualitatively different, and the structure exhibits a more stable behavior (Figure 6b). Both electron and hole trapping increase gradually up to about 1000 P/E cycles. The degradation of the endurance characteristics is observed for a larger number (>1000) of P/E cycles. *V_fb_* related both to electron and hole trapping decreases gradually as the number of P/E cycles exceeds 1000. The decrease related to positive charge trapping is stronger. Despite the decrease, the *V_fb_* related to electron trapping at about 1.5 × 10^4^ P/E cycles is still larger than that in the first P/E cycle. The evolution of the C-V curves with an increasing number of P/E cycles (Figure 7) gives evidence for a possible reason of the endurance degradation of annealed stacks.

The slope of the C-V curves changes with the P/E cycles, which reveals the interface states generation. These are most probably traps close to the valence band edge, which allow trapped holes to be easily discharged. Figure 7 also demonstrates that after positive charging of the capacitors (under −*V*_p_), some severe changes in the control C-V curve shape occur, which are not observed with electron injection (+*V*_p_). In the positively charged state, the region of the C-V curve corresponding to the transition from the flat band to inversion is characterized by a low slope. As this slanting section is not observed in the curves after electron injection, its origin is most probably not related to the interface states at the TO/Si interface. Although further investigation is required, we could suggest that this behavior is due to positive charge leakage through annealing defects in both *BO* and TO layers. As mentioned above, the interaction of the high-*k* stack with TO and *BO* during the RTA (evidenced by TEM) could be the reason for the deterioration of retention characteristics.

**Figure 8:**
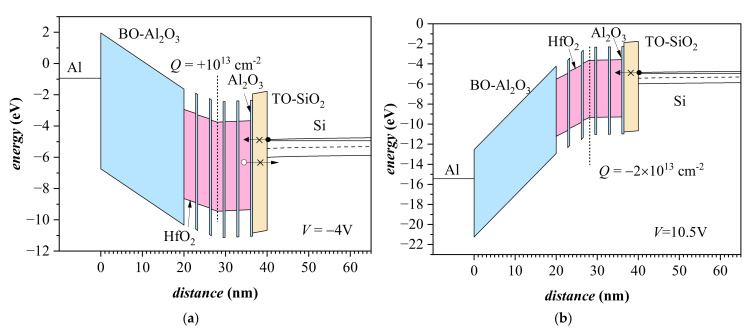
Band diagrams of capacitor with 3.5 nm SiO_2_ TO layer with positive trapped charge at applied voltage of −4 V (**a**), and (**b**) with negative trapped charge at *V* = 10.5 V. The trapped charge is roughly equal to the stored charges in the capacitor presented in Figure 7.

For illustration, Figure 8 demonstrates band diagrams of investigated capacitors with trapped positive (V = −4 V) and negative (V = 10.5 V) charge, respectively. Band diagrams are obtained with the Multi-Dielectric Energy Band Diagram Program [32]. In the case of positive trapped charge in weak and strong inversion (Figure 8a), some of the trapped holes could be lost by tunneling through TO facilitated by defect sites (denoted by x in Figure 8) either in the bulk of TO or at its interfaces with Si and high-*k* stack. Additionally, the positive trapped charge could also be decreased by the tunneling of electrons from the inversion layer via defect sites in TO into the HfO_2_-Al_2_O_3_ stack. When the trapped charge is negative, under inversion conditions, the tunneling electrons from Si will compensate the charge leakage toward the Al gate (Figure 8b); the tunneling of holes into the CT stack is hampered by their low density in the Si surface region.

## 4. Conclusions

The results presented in this work reveal that the charge trapping and storage in the metal/blocking oxide/high-k charge trapping layer/tunnel oxide/Si (MOHOS) structures with HfO_2_/Al_2_O_3_-based CTL are strong functions of the stack parameters (composition of CTL; type of tunneling oxide; thickness) as well as annealing steps. Electron trapping in HfO_2_/Al_2_O_3_ nanolaminate stacks is stronger compared to Al-doped HfO_2_ layers, while neither the retention of electrons, nor their discharge mechanism and discharge rate depend on the CTL. These results imply that electron trapping in both types of CTL occurs in the same type of traps, the density of which is higher in the HfO_2_/Al_2_O_3_ nanolaminate, and gives us a reason to conclude that these traps are related to Al_2_O_3_. On the other hand, hole trapping does not depend on the CTL; hence, it is related to HfO_2_ traps. The retention of both electrons and holes is most strongly affected by the tunneling oxide and its thickness. The 3.5 nm SiO_2_ provides a good barrier to back-tunneling of trapped charges. The as-deposited HfO_2_/Al_2_O_3_-based CTLs are vulnerable to high electric field stress, which generates a positive charge and deteriorates their endurance characteristics. The post-deposition O_2_ annealing significantly increases the electron trapping in the stacks and improves their susceptibility to high electric field stress, which manifests as wider memory windows and better endurance characteristics. However, high-temperature annealing deteriorates the retention of stored charges, which is most likely due to defects generation in the tunneling oxide as a result of the interfacial reaction between CTL and TO. Al_2_O_3_ deposited by ALD at low temperature is not suitable for the tunneling oxide. The results presented could help in a rational approach toward engineering of MOHOS structures to be implemented in flash NVM memories.

## Figures and Tables

**Table 1:** Spatial density of trapped electrons, *ρ*_e_, and holes, *ρ*_h_, for the structures annealed in O_2_.

Structure Type	*ρ*_e_ (cm^−3^)	*ρ*_h_ (cm^−3^)
*BO*/5 × (20:5)/2.4 nm SiO_2_	9.59 × 10^18^	1.48 × 10^19^
*BO*/5 × (20:5)/3.5 nm SiO_2_	1.45 × 10^19^	1.54 × 10^19^
*BO*/5 × (20:5)/3 nm Al_2_O_3_	3.60 × 10^18^	8.00 × 10^18^
*BO*/25 × (4:1)/2.4 nm SiO_2_	9.52 × 10^18^	1.29 × 10^19^
*BO*/25 × (4:1)/3.5 nm SiO_2_	1.58 × 10^19^	1.21 × 10^19^

## Data Availability

Not applicable.

## Supplementary Materials

The following supporting information can be downloaded at: https://www.mdpi.com/article/10.3390/ma15186285/s1, Figure S1: An illustration of the memory window definition.
