# Supplementary material for: Charge Storage and Reliability Characteristics of Nonvolatile Memory Capacitors with HfO2/Al2O3-Based Charge Trapping Layers
Source: Materials (Basel). 2022 Sep 9;15(18):6285. doi: 10.3390/ma15186285 (PMC9505722; doi:10.3390/ma15186285)
Supplement: Supplementary file 1 [file materials-15-06285-s001.zip › materials-1891628-supplementary.pdf]

Supplementary material

Article

Charge Storage and Reliability Characteristics of Nonvolatile Memory Capacitors with HfO_2/Al_2O_3-Based Charge Trapping Layers

Dencho Spassov [1], Albena Paskaleva [1,*], Elżbieta Guziewicz [2], Wojciech Wozniak [2], Todor Stanchev [1], Tsvetan Ivanov [1], Joanna Wojewoda-Budka [3] and Marta Janusz-Skuza [3]

[1] Institute of Solid State Physics, Bulgarian Academy of Sciences, Tzarigradsko Chaussee 72, 1784 Sofia, Bulgaria
[2] Institute of Physics, Polish Academy of Sciences, Al. Lotników 32/46, 02-668 Warsaw, Poland
[3] Institute of Metallurgy and Materials Science, Polish Academy of Sciences, ul. Reymonta 25, 30-059 Cracov, Poland
* Correspondence: paskaleva@issp.bas.bg

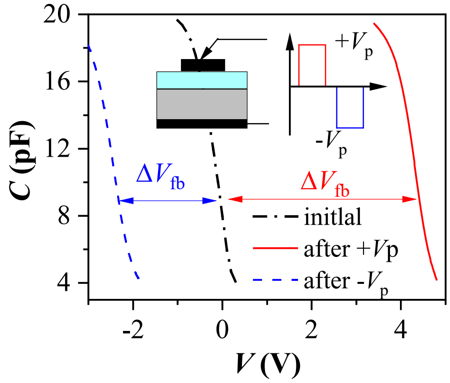

Figure S1. An illustration of the memory window definition. The memory window, ΔV is defined as the difference in the position of the measured C-V curves (measured flat band capacitance C_{fb}) after applying consecutively positive voltage pulse (+Vp), under which negative charges are injected from Si and trapped in CTL, and negative voltage pulse(–Vp), under which positive charges are injected and trapped in CTL . The inset shows the measurement procedure.
